# Advancing paediatric cardiac imaging: a comprehensive analysis of the feasibility and accuracy of a novel 3D paediatric transoesophageal probe

**DOI:** 10.3389/fcvm.2023.1294109

**Published:** 2023-12-05

**Authors:** Clément Karsenty, Khaled Hadeed, Pierrick Pyra, Aitor Guitarte, Camelia Djeddai, Remi Vincent, Yves Dulac, Ia Silagdze, Julie Gobin, Nicolas Combes, Miarisoa Ratsimandresy, Lionel Berthomieu, Davide Calvaruso, Philippe Acar

**Affiliations:** ^1^ Department of Paediatric Cardiology, University Hospital, Toulouse, France; ^2^ Institut Des Maladies Métaboliques Et Cardiovasculaires [Institute of Metabolic and Cardiovascular Diseases], University of Toulouse, Toulouse, France; ^3^ Department of Anesthesiology and Paediatric Cardiology, University Hospital, Toulouse, France; ^4^ Department of Cardiology, Clinique Pasteur [Pasteur Clinic], Toulouse, France; ^5^ Department of Intensive Care Unit and Paediatric Cardiology, University Hospital, Toulouse, France

**Keywords:** congenital heart disease, 3D echocardiography, transoesophageal echocardiography, pediatric, cardiac cateterism, pediatric cardiac surgery, complex congenital heart disease

## Abstract

**Aims:**

Pediatric transoesophageal echocardiography (TOE) probes have remained two-dimensional (2D) limiting their use compared to adults. While critical in pediatrics for interventions and post-surgery assessments, technological advancements introduced a three-dimensional (3D) pediatric TOE probe. This study assessed the new 3D pediatric TOE probe (GE 9VT-D) for feasibility, handling, and imaging quality.

**Methods and results:**

At Children's Hospital of Toulouse, 2-month prospective study enrolled children undergoing TOE with the new probe. All imaging modalities were rated by 2 operators using a 5-point Likert-type scale from 1 (very poor) to 5 (very good) quality. Forty-five children, median age 3.7 (range: 2 months-14.7 years) median weight 7.8 kg (range: 4.3–48 kg) underwent 60 TOEs: 25% pre-surgery, 45% post-surgery, 28% during percutaneous procedures, and 2% in intensive care. Probe handling was “very easy” in all cases without adverse events. The median score of 2D, 2D colour, pulsed Doppler and 3D were noted 5 out of 5 and continuous Doppler and 3D colour 4 out of 5. The 3D image quality remained consistent irrespective of the patient weighing above or below 7.8 kg (*p* = 0.72). Postoperative TOEs identified two cases needing further interventions, emphasizing its value in evaluating surgical outcomes and also for guiding percutaneous interventions.

**Conclusion:**

Our comprehensive evaluation demonstrates that the new 3D pediatric TOE probe is feasible and provides high-quality imaging in pediatric patients. The successful integration of this novel probe into clinical practice has the potential to enhance diagnostic accuracy and procedural planning, ultimately optimizing patient outcomes in pediatric cardiac care.

## Graphical Abstract

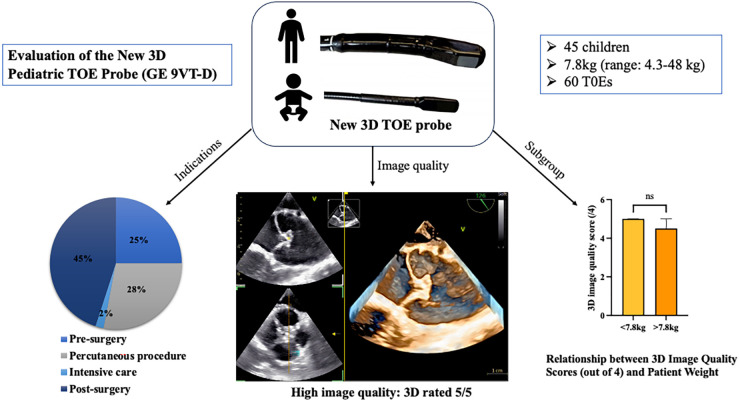

## Introduction

Real-time 3D echocardiography (3DE) has been made possible since the 1990 s, thanks to the development of matrix array probes featuring parallel processing capabilities. Advances in miniaturization have enabled the development of adult-size 3D transoesophageal echocardiography (TOE) probes (1).

The evolution of 3DE has revolutionized cardiac imaging, offering superior spatial resolution and improved visualization of complex cardiac structures, leading to enhanced diagnostic accuracy and therapeutic guidance (2). TOE is a crucial imaging modality for managing patients with congenital heart disease (CHD) (3). 3D TOE is primarily employed during surgical procedures for CHD to enhance diagnosis, identify residual lesions, and guide interventions in the cardiac catheterization laboratory (4, 5).

When considering a patient for TOE, their size and age are fundamental factors to consider. Most children typically undergo TOE under general anaesthesia, whereas conscious sedation is the norm for adult CHD patients (6). The selection of a suitable TOE probe primarily depends on the patient's weight. However, due to the larger size of 3D probes, 3D TOE was previously limited to older children and adolescents weighing >25–30 kg, thus restricting its use in the pediatric population (3).

Recent advancements in echocardiographic technology have spurred the development of a novel 3D pediatric TOE probe, designed to address the unique challenges associated with imaging young patients. Thus, a new 3D pediatric TOE probe (GE 9VT) received CE approval. This probe offers all imaging modes (from 2D to 3D) (7, 8). However, the successful integration of any new technology into routine clinical practice necessitates evaluation of its feasibility, accuracy, and safety.

Hence, the primary objective of our study was to assess the imaging quality of this innovative probe in a pediatric population. Additionally, we aimed to evaluate its safety and handling as secondary objectives.

## Methods

### Patients

Consecutive children were prospectively enrolled from the pediatric cardiology unit of the Children's Hospital of Toulouse during 2 months and half. Those requiring a TOE for medical reasons, such as cardiac surgery, interventional procedures, or intensive care unit (ICU) management were included in the study. Exclusion criteria were limited to cases where consent was not obtained.

Informed verbal consent was obtained from each patient, whenever feasible, and their legal representatives after providing a thorough explanation of the procedure.

### TOE probe insertion

The TOE probe was consistently inserted after patient sedation and intubation, prior to surgery or catheterization, by the anaesthesiologist. In the ICU, the TOE was utilized in mechanically ventilated patients when transthoracic echocardiography was insufficient. The pre-lubricated probe was gently and blindly inserted with a jaw thrust of the mandible or, if not feasible, under direct visualization by laryngoscopy. Following the initial examination during CHD surgeries, the probe was further advanced into the stomach and left in an unlocked position throughout the procedure. Subsequently, the post-repair TOE examination was performed prior to discontinuing cardiopulmonary bypass.

### TOE image acquisition and scoring system

For CHD surgery, TOE examinations were conducted post-surgery to assess residual lesions, and sometimes performed pre-surgery to validate or adjust preoperative diagnosis. These examinations were performed by one of two primary operators (K. H. or P. P.) following international guidelines (5). Subsequently, off-line analysis was carried out by two operators (C. K. or K. H.), who evaluated the image quality. Inter-rater agreement of the 3D imaging quality was also assessed. Additionally, the ease of use (handling) of the probe was scored by the operators.

The quality of each modality was evaluated using a 5-point Likert-type scale for each category (e.g., 5 = very good image quality; 4 = good image quality 3 = average image quality; 2 = poor quality and 1 = very poor quality). Insertion and handling were also rated using a 5-point Likert-scale (5 = very easy, 4 = easy, 3 = average, 2 = difficult, 1 = very difficult).

Image quality parameter such as “imaging frequency (f)” that influences spatial resolution and “volumetric frame rate (VFR)” that affects temporal resolution were provided for 3D data set. The compression was 63 decibels for all acquisitions. For the first 10 exams we used both multi-beat and single-beat acquisition and then only single-beat real-time acquisition without ECG gating. We compared the quality imaging between single-beat and multi-beat 3D acquisition.

Imaging was obtained via GE system, E95 (GE), with the new GE 9VT 3D TOE probe.

### Statistical analysis

Age, weight and image quality are expressed as median [range or first interquartile range (IQR), third interquartile range]; qualitative data are expressed as number and percentage. Comparisons of ratings between the groups were performed using a non-parametric Mann–Withney test. A subgroup analysis was assessed according to weight of patients and the cut-off value of weight was the median weight of the population. Correlation between 3D quality and f and VFR were assessed using a nonparametric Spearman test. Interrater agreement between the two operators was assessed using the kappa coefficient for 3D quality of imaging. A *p*-value <0.05 was considered statistically significant. Statistical analysis was performed using Graphpad Pirsm v8.

## Results

A total of 45 children, including 23 females and 22 males (median [range] age 3.7 years [2 month—14.7 years]; weight 13.0 [4.3–48] kg) underwent 60 TOE examinations using the 9VT 3D TOE probe. TOE indications, along with numbers of TOEs per patient, are shown in Table 1.

**Table 1:** Indications for the transoesophageal echocardiography (TOE) examinations.

	*n *= 60
TOE indications	
Presurgical assessment	15
Postsurgical assessment	27
Percutaneous procedures	17
ICU	1
Surgeries	
AVSD	12
ASD	2
VSD	15
Sub-aortic membrane	4
Pezzy-Laubry syndrome	2
Conotruncal cardiopathy	7
Mitral valve repair	3
Prosthetic pulmonary tube endocarditis	2
Multistage pulmonary stenosis	1
Percutaneous procedures	
ASD	9
VSD^a^	6
PFO	1
Diagnostic in Ebstein	1
*ICU*	
HCM	1
Number of examinations per patient	
1	28
2	13
3	2

ASD, atrial septal defect; AVSD, atrioventricular septal defect; HCM, hypertrophic cardiomyopathy; ICU, intensive care unit; VSD, ventricular septal defect; PFO, patent foramen ovale.

^a^ 2 coarctation syndrome with pulmonary artery bandage.

### Image Quality Evaluation

**Figure 1:**
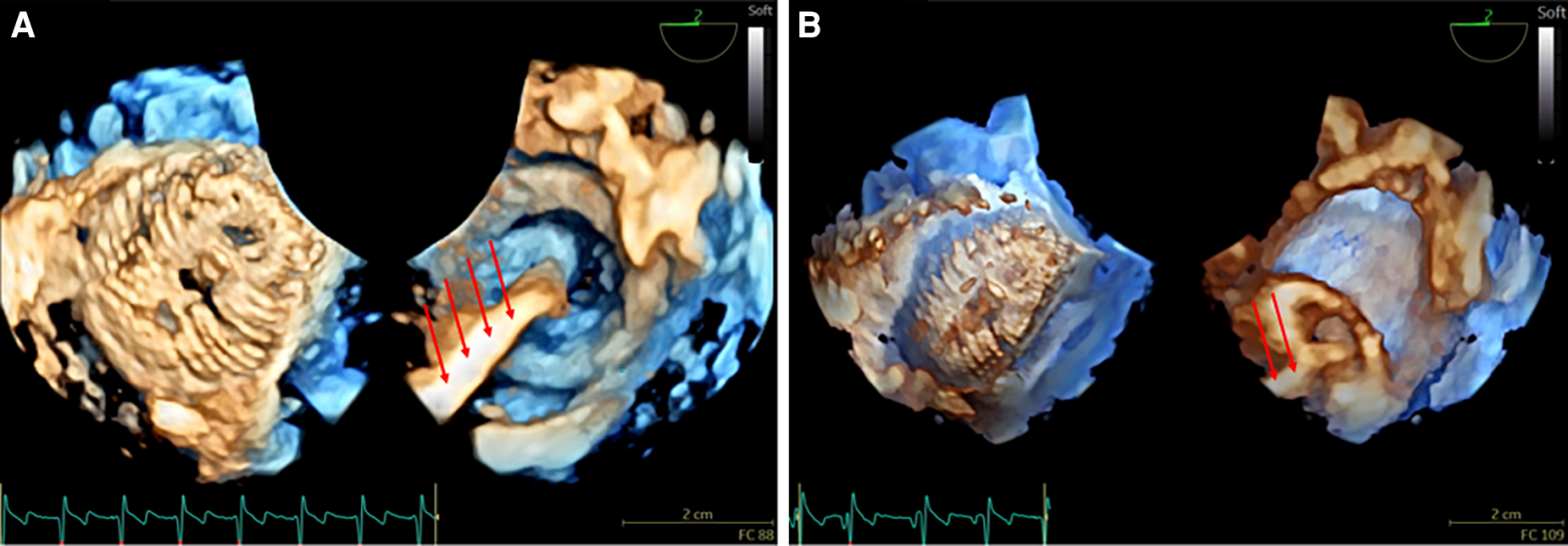
3D real-time TOE of the atrial septal device before release in a 17 kg patient with a central atrial septal defect (ASD). (**A**) The figure demonstrates a 3D en face view of a 22 mm device from both the left atrium (left panel) and the right atrium (right panel) with the wire still attached (red arrows) (Supplementary Video S1). (**B**) Depiction of the “Minnesota maneuver” showing the pull on the right disk (Supplementary Video S2). 3D: three-dimensional; ASD, atrial septal defect; LA, left atrium; RA, right atrium; TOE, transoesophageal echocardiography.

**Figure 2:**
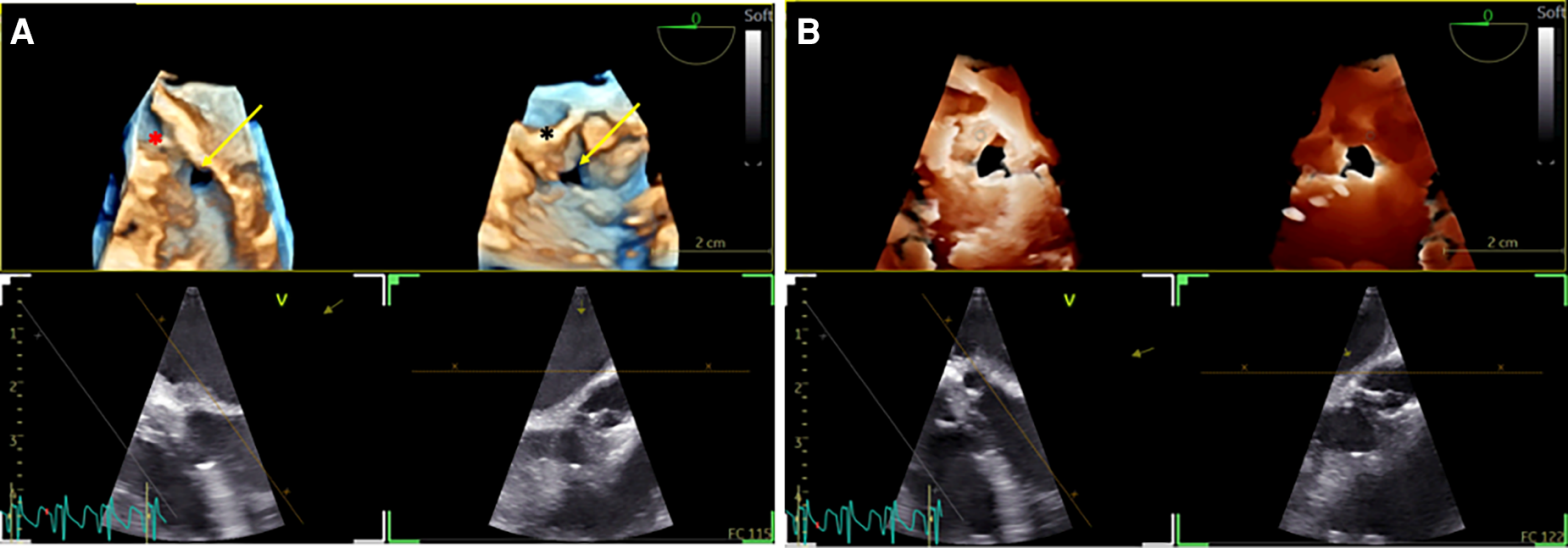
3D TOE in a 5 kg patient with a ventricular septal defect (VSD). (**A**) Dual cropping views on 3D real-time of the perimembranous VSD with outlet extension (yellow arrow) from the LV close to the aortic valve (red asterix) (left panel) and from the RV close to the tricuspid valve (black asterix) (Supplementary Video S3). (**B**) Transillumination rendering in dual cropping views with Flexilight™ highlighting the perimembranous VSD with outlet extension (Supplementary Video S4). 3D, three-dimensional; LV, left ventricle; RV, right ventricle; VSD, ventricular septal defect.

**Figure 3:**
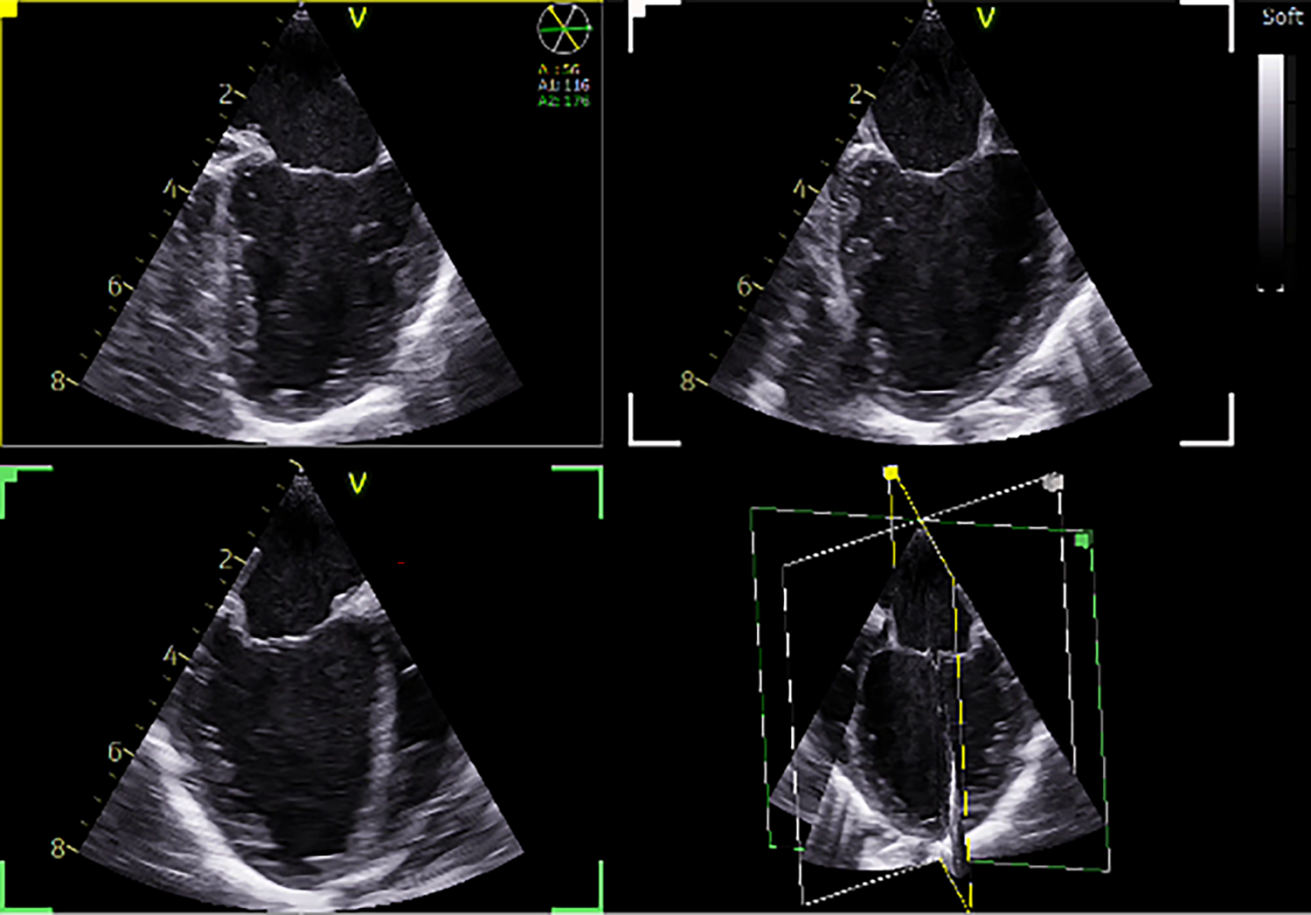
Tri-plane real-time left ventricular (LV) view. Demonstration of normal LV function in a 5 kg patient with a VSD (Supplementary Video S5). The same patient as in Figure 3. VSD: Ventricular septal defect.

**Figure 4:**
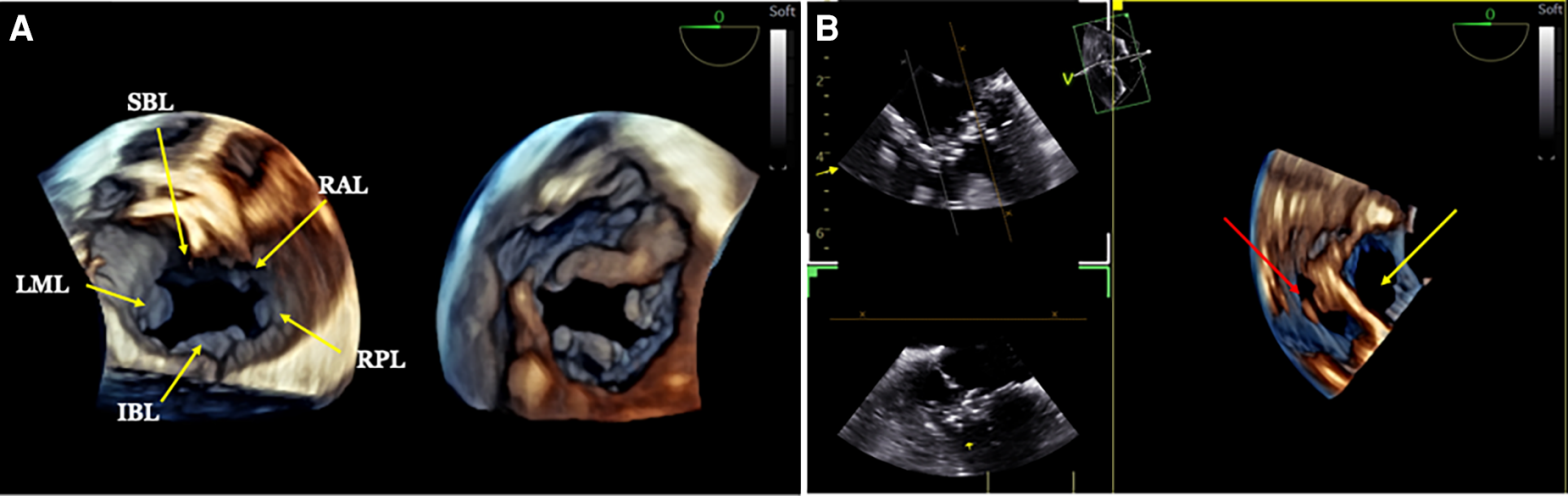
3D TOE in a 5.2 kg patient with a complete atrioventricular septal defect (AVSD). (**A**) Dual cropping views on 3D real-time of the common valve from the atrium (left panel), displaying the five leaflets, and from the ventricle (right panel) (Supplementary Video S6). (**B**) 3D live view from the lateral aspect of the LV showing both the ostium primum ASD (yellow arrow) and the inlet VSD (red arrow) (Supplementary Video S7). 3D, three-dimensional; AVSD, atrioventricular septal defect; ASD, atrial septal defect; LV, left ventricle; VSD, ventricular septal defect; SBL, superior bridging; RAL, right anterior leaflet; LML, left mural leaflet; IBL, inferior binding leaflet; RPL, right posterior leaflet.

**Figure 5:**
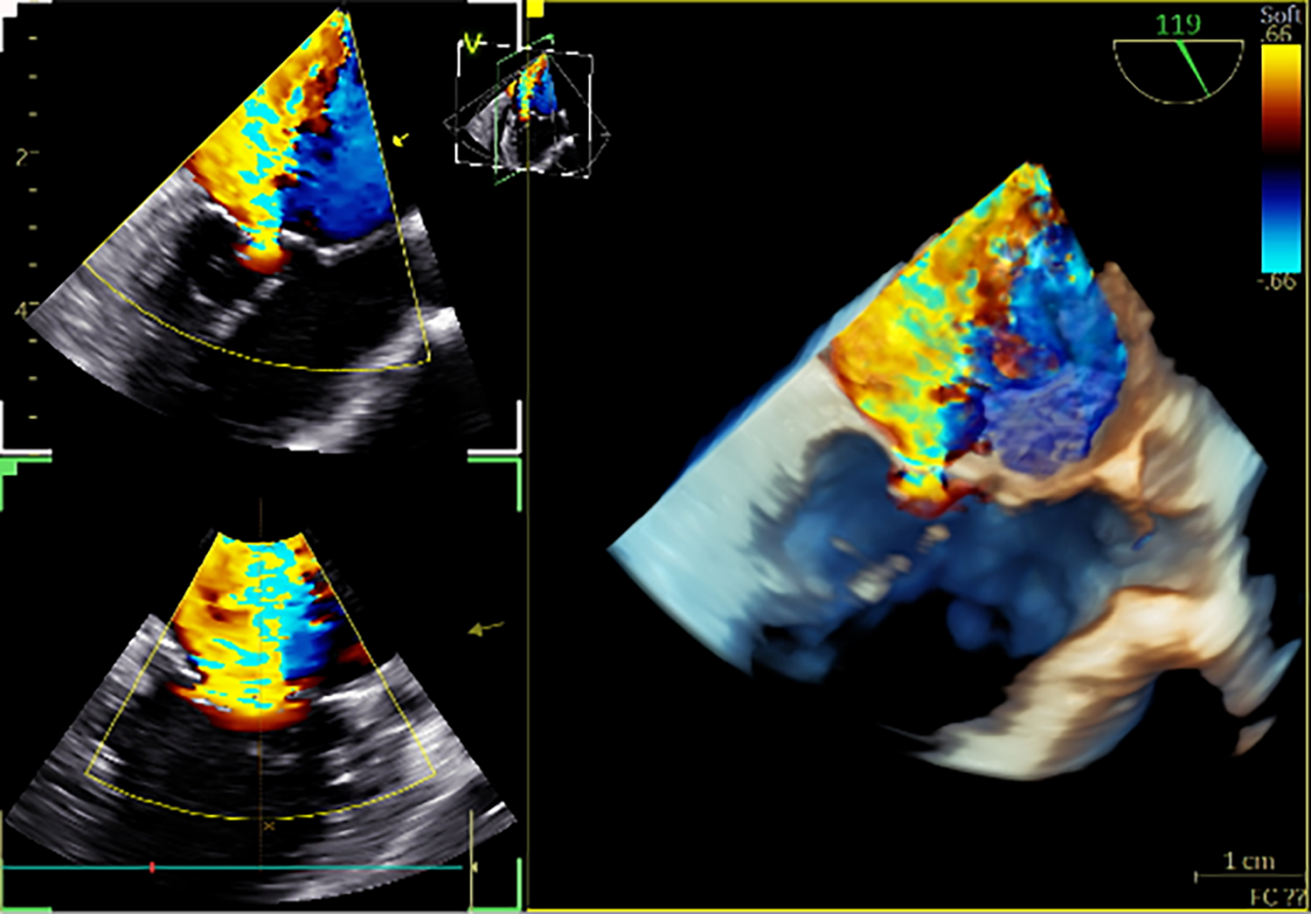
3D colour TOE in a 9.6 kg patient with mitral valve regurgitation. The figure presents a 3D colour live view of severe functional mitral regurgitation without any congenital abnormality of the valve (Supplementary Video S8). 3D, three-dimensional.

During surgeries and cardiac catheterization, 3D images obtained from the 9VT 3D TOE probe were rated “average” [*n* = 6 (10%)], “good” [*n* = 22 (37%)] or “very good” [*n* = 32 (53%)] quality. All quality image results according to echocardiographic modality are presented in Table 2 and highlighted in Figures 1–5 and Supplementary Videos S1–S8). Inter-rater agreement for 3D image quality evaluation demonstrated strong concordance (weighted kappa coefficient 0.92, *p* < 0.0001).

**Table 2:** Image quality results according to echocardiographic modality.

Echocardiographic Modality	Number of Examinations	Very Good (Score 5)	Good (Score 4)	Average (Score 3)	Poor (Score 2)	Very Poor (Score 1)
2-dimensional	60	56	4	0	0	0
2D colour	60	54	5	1	0	0
Pulsed Doppler	47	36	8	2	1	0
Continuous Doppler	51	32	15	3	0	1
3-dimensional	60	32	22	6	0	0
3D colour	47	16	20	9	2	0

Image quality scores are rated on a 5-point Likert-type scale, with 5 being “Very Good,” 4 being “Good,” 3 being “average”, 2 being “Poor” and 1 being “Very Poor.” The number of examinations with each image quality rating is presented for each echocardiographic modality.

Median [range] f was 5 [5–8] MHz and median VFR was 31 [19–48] and weren't correlated with 3D quality image (*r* = 0.10, *p* = 0.64; and *r* = −0.30, *p* = 0.17 respectively). No difference in image quality was found between single-beat and multi-beat 3D acquisition (*p* = 0.20). Furthermore, motion artifact was observed for two exams leading to a decrease in image quality in multi-beat acquisition.

### Subgroup analysis

Subgroup analysis based on patients' weight, did not reveal any significant impact on the image quality score of the 3D TOE probe (*p* = 0.72).

**Figure 6:**
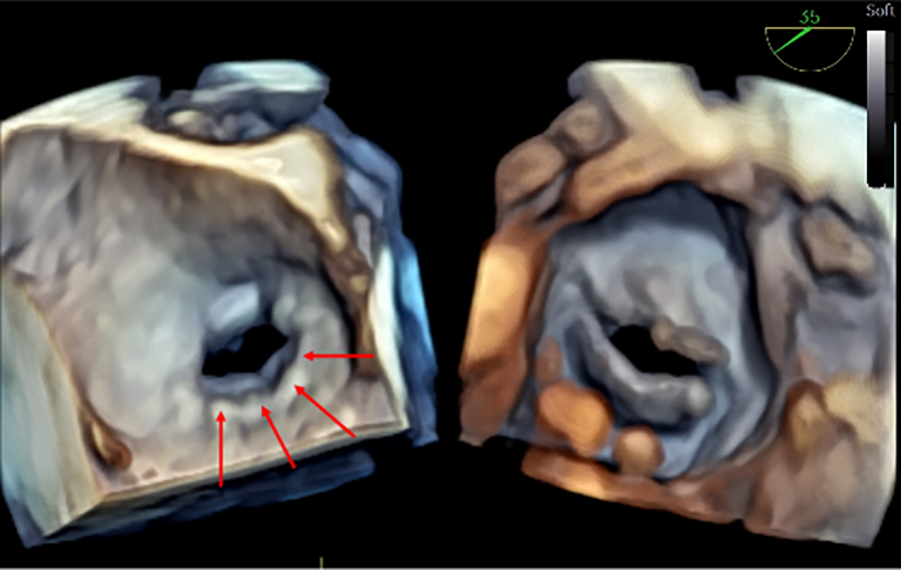
3D TOE in a 37 kg patient with mitral stenosis. The figure displays dual cropping views on 3D real-time of the mitral valve. From the left atrium (left panel), a supravalvular ring is visible (red arrows), and from the left ventricle (right panel) (Supplementary Video S9). 3D, three-dimensional; LV, left ventricle.

**Figure 7:**
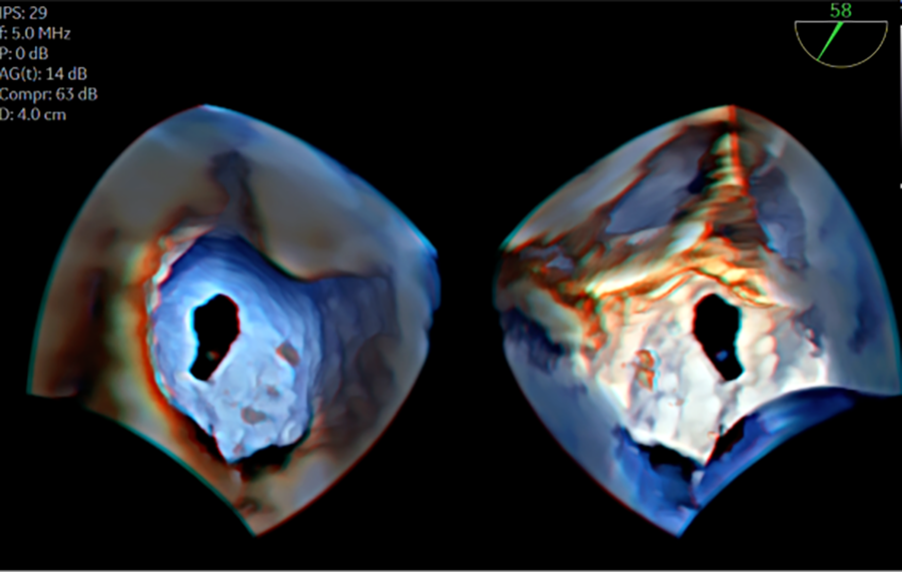
3D TOE in a 18 kg patient with ostium secundum ASD. Transillumination rendering in dual cropping views with FlexilightTM showing en face view of the ASD from the RA (left panel) and the LA (Supplementary Video S10). 3D, three-dimensional; ASD, atrial septal defect; LA, left atrium; RA, right atrium; TOE, transoesophageal echocardiography.

**Figure 8:**
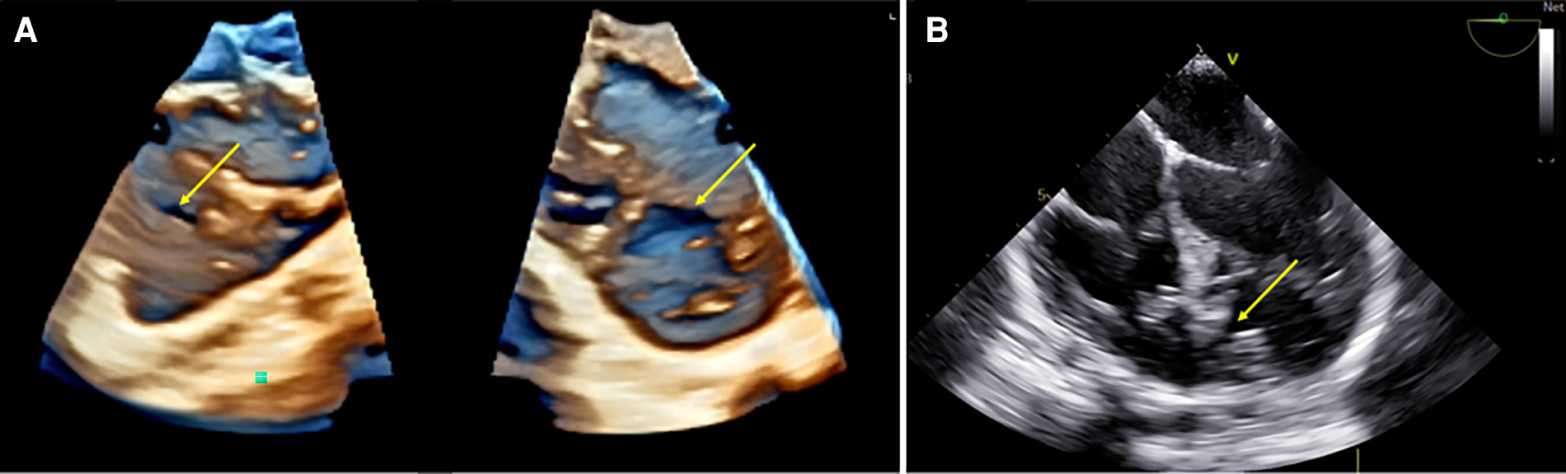
3D TOE in a 15 kg patient with muscular VSD. (**A**) Dual cropping views on 3D real-time of the midseptum muscular VSD (yellow arrow) from the LV (right panel) and from the RV with a typical oval shape (Supplementary Video S11). (**B**) 2D view of the VSD (yellow arrow) showing the limit of the 2D assessment for size and shape. 3D, threedimensional; LV, left ventricle; RV, right ventricle; VSD, ventricular septal defect.

**Figure 9:**
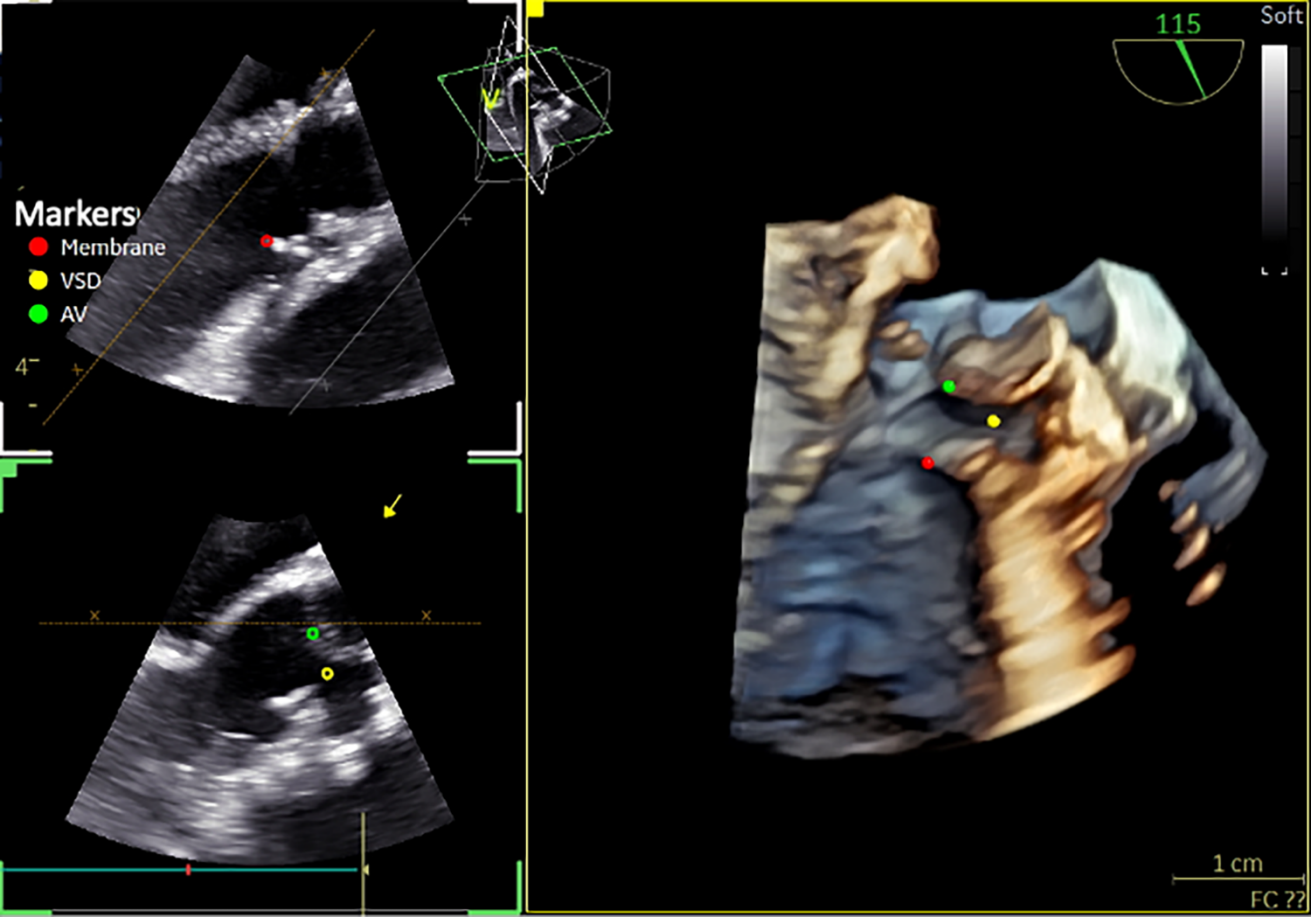
3D TOE in a 11 kg patient with VSD and subaortic membrane. Utility of markers positioned in 2D and visualized in 3D image (from the LV) showing the semi-circular membrane and the relationship with the VSD and the aortic valve (AV) (Supplementary Video S12). 3D, three-dimensional; LV, left ventricle; VSD, ventricular septal defect.

**Figure 10:**
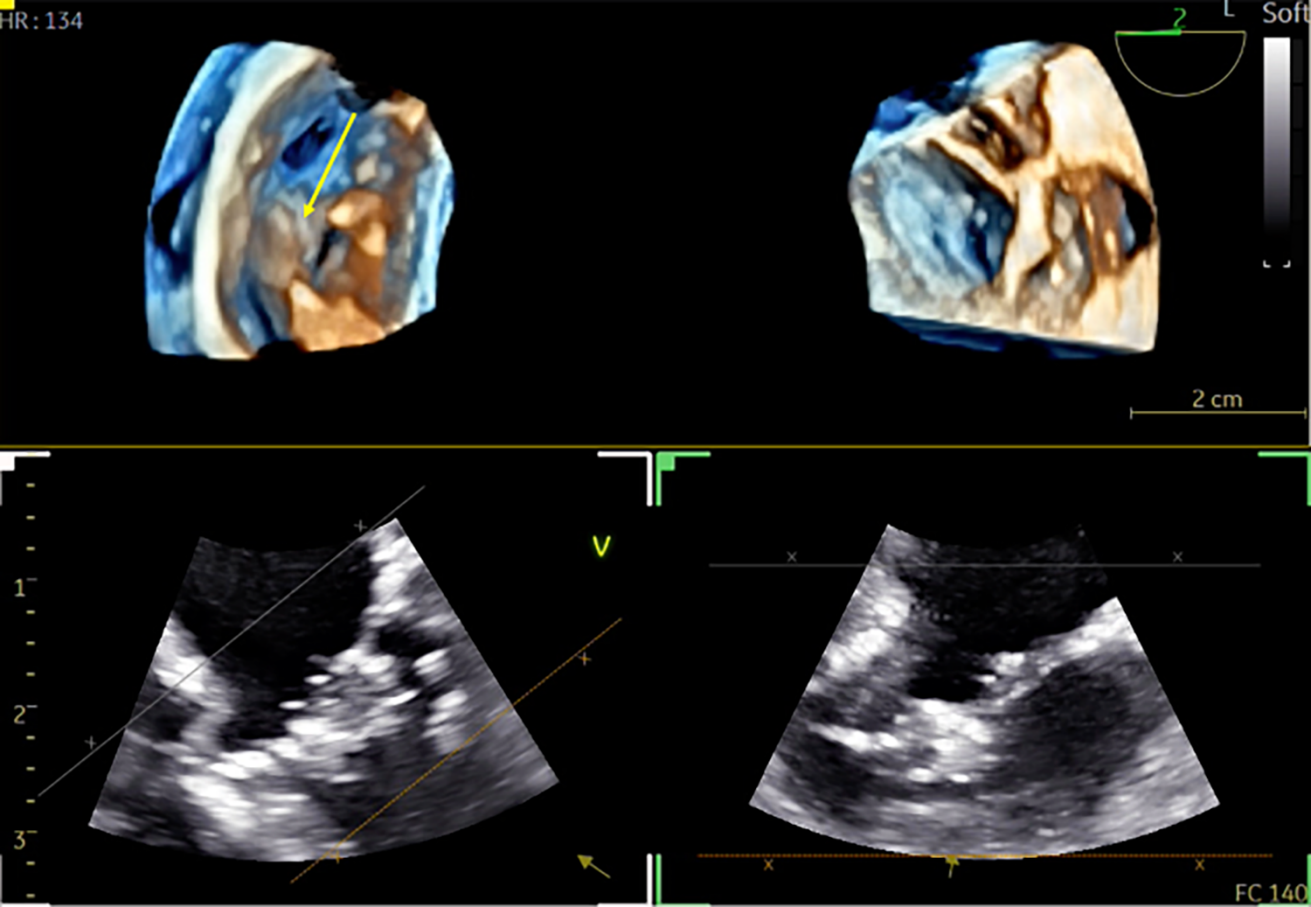
3D TOE in a 5.8 kg patient with repaired intermediate atrioventricular septal defect. Dual cropping views on 3D real-time of the left atrioventricular valve with the cleft closure (yellow arrow) from the LV (left panel) (Supplementary Video S13). 3D, three-dimensional; LV, left ventricle.

**Figure 11:**
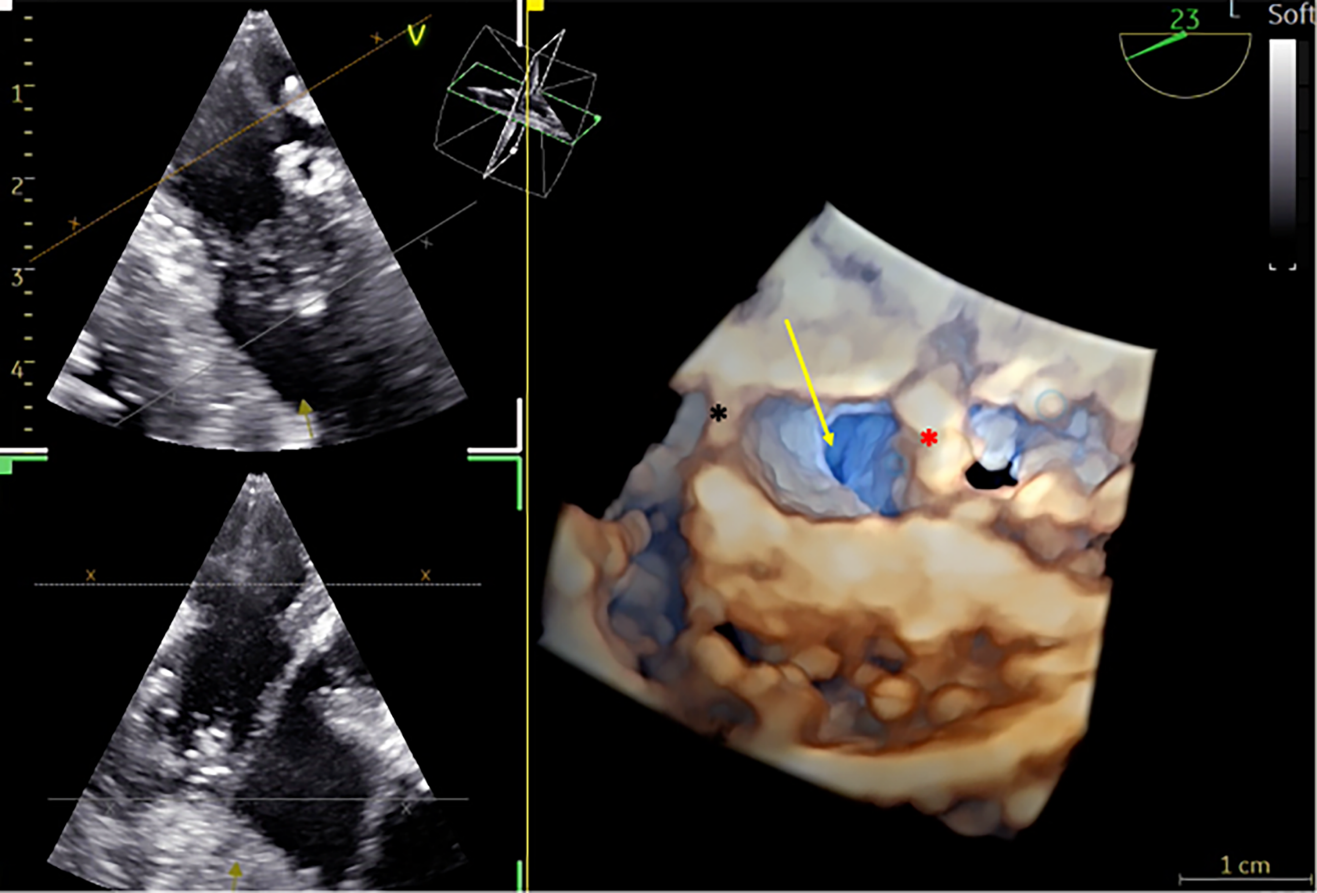
3D TOE in a 7.8 kg patient after modified Konno procedure. 3D view showing the massive relief (yellow arrow) and the interventricular septum patch (black asterix) and the relationship with the anterior mitral valve (red asterix) (Supplementary Video S14).

Clinical impact in 14 examinations (out of 60): In two patients, reintervention was needed due to severe residual regurgitation of the left atrioventricular valve, as visualized on TOE. Additionally, in one patient, the diagnosis was corrected from mitral valve stenosis to supravalvular membrane, thanks to the enhanced visualization provided by 3D images (Figure 6 and Supplementary Video S9). Six patients underwent percutaneous closure of ostium secundum atrial septal defect (ASD). The 3D provides an en-face view of the ASD with a comprehensive view of the surrounding edges at a single glance (Figure 7 and Supplementary Video S10). In 2 patients, the ventricle septal defect (VSD) was better delineated with 3D, having a slit-like shape, allowing for a more appropriate choice of the percutaneous prosthesis (Figure 8 and Supplementary Video S11). One subaortic membranous was better defined by 3D having a semi-circular shape which helps the surgeon (Figure 9 and Supplementary Video S12). In 2 cases TOE improves postoperative understanding: (1) After cleft closure in intermediate atrioventricular septal defect the 3D image displayed very well the sutured cleft which is not visible in 2D images (Figure 10 and Supplementary Video S13); (2) After a modified Konno (to release the obstruction in hypertrophic cardiomyopathy) the muscular septal resection with the interventricular septum patch and its relationship with the anterior mitral valve was clearly defined by 3D images (Figure 11 and Supplementary Video S14).

### Feasibility of probe insertion

Successful insertion of the 9VT 3D TOE probe was achieved in all cases without encountering any significant technical difficulties, and handling was rated as very easy for all patients (rated 5/5).

#### Safety and adverse events

No major adverse events related to the use of the 3D pediatric TOE probe were observed during the study period.

## Discussion

Our findings demonstrate that the new 3D pediatric TOE probe offers a promising advancement in pediatric cardiac imaging. The probe's high feasibility and successful insertion rate in all enrolled patients indicate its safety and efficiency in the pediatric population. The primary focus of our investigation was to assess the image quality and diagnostic utility of the 3D TOE probe. Our results show that the probe consistently produced high-quality 2D and 3D images, with all rated as “good” or “very good”. This demonstrates the probe's ability to provide clear and precise visualization of cardiac structures, allowing for improved assessment and diagnosis of CHD lesions and to guide intervention.

Our study also emphasized the probe's diagnostic accuracy across different age and weight groups, including neonates, infants, and older children. The 3D pediatric TOE probe consistently demonstrated excellent image quality, irrespective of patient weight, making it a versatile imaging tool in pediatric cardiology.

Before the release of this new 3D pediatric TOE probe, intraoperative epicardial 3D imaging using a transthoracic 3DE transducer offers a feasible alternative technique during surgery (9). Prior studies have demonstrated the feasibility of obtaining high-quality diagnostic 3D en face images of ventricular septal defects (VSD) using 3D epicardial echocardiography in a patient cohort ranging from 5 to 20 kg (10). However, one of the challenges hindering the routine adoption of intraoperative 3D epicardial echocardiography is the steep learning curve during the initial phase. Additionally, effective communication between the cardiac sonographer and the operating surgeon is crucial for appropriate probe placement on the heart without compromising hemodynamic stability.

On the other hand, micro-multiplane TOE probe routinely used for perioperative study, allow 2D images in children weighting more than 3 kg with good quality (11). However, the image quality decrease with increasing patient weight and 2D quality remains lower than in our study, and micro-TOE probe lacked 3D technology.

Interestingly, we didn't find a significant difference in image quality between multi-beat and single-beat acquisition without ECG gating simplifying the use of 3D in everyday life.

In the catheterization laboratory, 3D TOE offers precise measurements of cardiac structures, facilitating appropriate device selection and reducing the risk of procedural complications (12). 3D TOE offers real-time imaging with continuous monitoring of catheter-based interventions. The ability to visualize devices and catheters in 3D allows interventional cardiologists to guide their placement with greater accuracy. In parallel, the fusion of 3DE and fluoroscopy in the catheterization laboratory is becoming more common, but until is currently only compatible with the matrix adult TOE probe. In fusion imaging, the TOE transducer position and orientation are automatically synchronized and co-registered with the fluoroscopy image. Additionally, markers can be applied to important structures on the 3DE images that are then simultaneously displayed on fluoroscopy, thus aiding in interventions. This tool would be helpful for pediatric complex CHD intervention below 30 kg (13, 14). This may reduce the need for repeated catheter manipulations and fluoroscopy, minimizing radiation exposure to pediatric patients.

Furthermore, beyond enhancing precision and care through 3D TOE, the utilization of 3DE may improve education and communication among practitioners and with families, particularly when employing 3D printed models (15, 16). Although CT images have been preferred to create 3D printed models because of their high spatial resolution, improvements in 3DE images resolution now also allow for detailed 3D printed models obtained from 3DE examinations (17, 18). However, these models are static, and they either exclude the valves or represent them in a static manner. Recent advancements in virtual reality/augmented reality offer novel avenues to augment the utility of 3D echocardiography. The integration of augmented reality with 3D echocardiographic imaging allows cardiologists and surgeons to immerse themselves within the heart's anatomy, facilitating a clearer understanding of complex structures and pathologies and aids in precise procedural planning and guiding (19, 20).

Substantially, even in the heaviest patient in our series (48 kg), the image quality remained excellent. It would be worthwhile to assess the probe in the adult population to determine its potential for broader use. Indeed, in adult patients, the new 3D pediatric TOE probe may have several advantages especially in the field of interventional cardiology (21). Its smaller size and flexibility enable easier insertion through the oesophagus, improving patient comfort during the procedure that may be performed without sedation (22). The miniaturization also opens up imaging possibilities for patients with restricted oesophageal access, such as those with a history of upper gastrointestinal surgery or anatomical anomalies (5).

### Limitations

While our study highlights the potential of the 3D pediatric TOE probe, certain limitations must be acknowledged. The sample size of the study and the potential for selection bias may limit the generalizability of the findings to a broader pediatric population. Nevertheless, our study included children of different weights with diverse indications for TOE, allowing for the evaluation of the new 3D pediatric probe in various clinical settings.

Moreover, it's pertinent to recognize that our study did not evaluate, and thus did not establish the superiority of 3D over 2D TOE. Although 3DE has been increasingly used in clinical practice due to its ability to provide additional diagnostic information, there is currently a lack of randomized trials relating to procedural success, morbidity, or mortality specifically related to the application of 3DE in pediatric patients. The adoption of 3DE in practice has largely been driven by the clinical need to enhance diagnostic capabilities rather than direct comparative evidence (23). However, we still managed to highlight an added value of 3D over 2D for 14 exams. Additionally, the need for specialized training and expertise in performing 3D TOE procedures in children may limit widespread adoption (24). We also acknowledge that not all 60 examinations assessed every image modality, which could be a potential limitation of our study.

And to finish a limitation is that we were not blinded in the analysis of image quality since we knew the images came from the new 3D probe under study. However, since this is the inaugural pediatric 3D probe, we cannot assess pediatric 3D TOE without being aware that they come from this unique probe offering pediatric 3D imaging.

## Conclusion

Overall, the results of this comprehensive analysis demonstrate that the new 3D pediatric TOE probe provide high-quality images and is a valuable advancement in pediatric cardiac imaging. Its high feasibility, accuracy, and safety make it a transformative tool in the evaluation and management of CHD across various patient profiles. These promising results encourage further research and clinical implementation to fully harness the benefits of this innovative imaging tool in pediatric cardiology.

### Clinical impact

The integration of the new 3D pediatric TOE probe into routine clinical practice appears to have a substantial impact on the management of pediatric patients with CHD. The reported enhancements in image quality and diagnostic accuracy suggest potential benefits for decision-making, patient care, and procedural outcomes. However, further research and comprehensive clinical studies are warranted to fully validate these observations and establish the precise extent of its influence in pediatric cardiology practice.

## Data Availability

The raw data supporting the conclusions of this article will be made available by the authors, without undue reservation.

## References

[1] Handke M Heinrichs G Moser U Hirt F Margadant F Gattiker F Transesophageal real-time three-dimensional echocardiography: methods and initial in vitro and human in vivo studies. J Am Coll Cardiol. (2006) 48:2070–6. 10.1016/j.jacc.2006.08.013 17112996

[2] Faletra FF Agricola E Flachskampf FA Hahn R Pepi M Ajmone Marsan N Three-dimensional transoesophageal echocardiography: how to use and when to use-a clinical consensus statement from the European association of cardiovascular imaging of the European society of cardiology. Eur Heart J Cardiovasc Imaging. (2023) 24:e119–97. 10.1093/ehjci/jead090 37259019

[3] Simpson J Lopez L Acar P Friedberg M Khoo N Ko H Three-dimensional echocardiography in congenital heart disease: an expert consensus document from the European association of cardiovascular imaging and the American society of echocardiography. Eur Heart J Cardiovasc Imaging. (2016) 17:1071–97. 10.1093/ehjci/jew172 27655864

[4] Madriago EJ Punn R Geeter N Silverman NH. Routine intra-operative trans-oesophageal echocardiography yields better outcomes in surgical repair of CHD. Cardiol Young. (2016) 26:263–8. 10.1017/S1047951115000098 25730612

[5] Puchalski MD Lui GK Miller-Hance WC Brook MM Young LT Bhat A Guidelines for performing a comprehensive transesophageal echocardiographic: examination in children and all patients with congenital heart disease: recommendations from the American society of echocardiography. J Am Soc Echocardiogr. (2019) 32:173–215. 10.1016/j.echo.2018.08.016 30579694

[6] Coisne A Dreyfus J Bohbot Y Pelletier V Collette E Cescau A Transoesophageal echocardiography current practice in France: a multicentre study. Arch Cardiovasc Dis. (2018) 111:730–8. 10.1016/j.acvd.2018.03.014 30539734

[7] Karsenty C Hadeed K Acar P. First experience with 3-dimensional pediatric transesophageal echocardiography. Rev Esp Cardiol. (2023) 76:487. 10.1016/j.recesp.2022.11.003 36427788

[8] Acar P Hadeed K Vignaud P Pyra P Guitarte A Dulac Y Such a long wait: three-dimensional paediatric transoesophageal echocardiography finally arises. Arch Cardiovasc Dis. (2023) 116:1–2. 10.1016/j.acvd.2022.10.002 36529646

[9] Muhiudeen IA Roberson DA Silverman NH Haas G Turley K Cahalan MK. Intraoperative echocardiography in infants and children with congenital cardiac shunt lesions: transesophageal versus epicardial echocardiography. J Am Coll Cardiol. (1990) 16:1687–95. 10.1016/0735-1097(90)90320-O 2254554

[10] Pillai MN Suneel PR Menon S Unnikrishnan KP Baruah SD Mathew T Intraoperative three-dimensional imaging of ventricular septal defects in children using epicardial echocardiography: a novel approach. J Cardiothorac Vasc Anesth. (2021) 35:2892–9. 10.1053/j.jvca.2020.10.051 33234468

[11] Hascoët S Peyre M Hadeed K Alacoque X Chausseray G Fesseau R Safety and efficiency of the new micro-multiplane transoesophageal probe in paediatric cardiology. Arch Cardiovasc Dis. (2014) 107:361–70. 10.1016/j.acvd.2014.05.001 24996565

[12] Hascoet S Hadeed K Marchal P Dulac Y Alacoque X Heitz F The relation between atrial septal defect shape, diameter, and area using three-dimensional transoesophageal echocardiography and balloon sizing during percutaneous closure in children. Eur Heart J Cardiovasc Imaging. (2015) 16:747–55. 10.1093/ehjci/jeu316 25617028

[13] Hadeed K Hascoët S Karsenty C Ratsimandresy M Dulac Y Chausseray G Usefulness of echocardiographic-fluoroscopic fusion imaging in children with congenital heart disease. Arch Cardiovasc Dis. (2018) 111:399–410. 10.1016/j.acvd.2018.03.006 29853351

[14] Hascoët S Hadeed K Karsenty C Dulac Y Heitz F Combes N Feasibility, safety and accuracy of echocardiography-fluoroscopy imaging fusion during percutaneous atrial septal defect closure in children. J Am Soc Echocardiogr. (2018) 31:1229–37. 10.1016/j.echo.2018.07.012 30219347

[15] Karsenty C Guitarte A Dulac Y Briot J Hascoet S Vincent R The usefulness of 3D printed heart models for medical student education in congenital heart disease. BMC Med Educ. (2021) 21:480. 10.1186/s12909-021-02917-z 34496844 PMC8424617

[16] Karsenty C Hadeed K Djeddai C Lateyron J Guitarte A Vincent R Impact of 3D-printed models in meetings with parents of children undergoing interventional cardiac catheterisation. Front Pediatr. (2022) 10:947340. 10.3389/fped.2022.947340 36699296 PMC9869040

[17] Vukicevic M Mosadegh B Min JK Little SH. Cardiac 3D printing and its future directions. JACC Cardiovasc Imaging. (2017) 10:171–84. 10.1016/j.jcmg.2016.12.001 28183437 PMC5664227

[18] Mowers KL Fullerton JB Hicks D Singh GK Johnson MC Anwar S. 3D echocardiography provides highly accurate 3D printed models in congenital heart disease. Pediatr Cardiol. (2021) 42:131–41. 10.1007/s00246-020-02462-4 33083888

[19] Pushparajah K Chu KYK Deng S Wheeler G Gomez A Kabir S Virtual reality three-dimensional echocardiographic imaging for planning surgical atrioventricular valve repair. JTCVS Techniques. (2021) 7:269–77. 10.1016/j.xjtc.2021.02.044 34100000 PMC8169455

[20] Chessa M Van De Bruaene A Farooqi K Valverde I Jung C Votta E Three-dimensional printing, holograms, computational modelling, and artificial intelligence for adult congenital heart disease care: an exciting future. Eur Heart J. (2022) 43:2672–84. 10.1093/eurheartj/ehac266 35608227

[21] Wang DD Forbes TJ Lee JC Eng MH. Echocardiographic imaging for left atrial appendage occlusion: transesophageal echocardiography and intracardiac echocardiographic imaging. Interv Cardiol Clin. (2018) 7:219–28. 10.1016/j.iccl.2018.01.001 29526290

[22] Sanchis L Regueiro A Cepas-Guillén P Sitges M Freixa X. First experience of left atrial appendage occlusion using a 3D mini transoesophageal echocardiographic probe with conscious sedation. EuroIntervention. (2023) 18:1460–1. 10.4244/EIJ-D-22-00921 36705913 PMC10111133

[23] Lang RM Addetia K Narang A Mor-Avi V. 3-Dimensional echocardiography: latest developments and future directions. JACC Cardiovasc Imaging. (2018) 11:1854–78. 10.1016/j.jcmg.2018.06.024 30522687

[24] Hahn RT Mahmood F Kodali S Lang R Monaghan M Gillam LD Swaminathan M Bonow RO. Core competencies in echocardiography for imaging structural heart disease interventions. JACC Cardiovasc Imaging 2019;12:2560–70. 10.1016/j.jcmg.2019.10.008 31806184 PMC7988896

